# Clinical Lecture on the Semeiology of the Eye*It is possible that the phraseology of the following lecture, prepared some time since, may, in a few instances, have been borrowed from other sources, and not marked as quotations at the time; and the authorities have now escaped from recollection. This statement is made to obviate any charge of plagiarism.

**Published:** 1850-07

**Authors:** Charles A. Lee

**Affiliations:** Prof. of Materia Medica, and Pathology, in the Medical Department of the University of Buffalo


### BUFFALO MEDICAL JOURNAL

AND

### MONTHLY REVIEW.

VOL. 6. JULY, 1850. NO. 2.

### ORIGINAL COMMUNICATIONS.

---

ART. I.—*Clinical Lecture on the Semeiology of the Eye.* By CHARLES A. LEE, M. D., Prof. of Materia Medica, and Pathology, in the Medical Department of the University of Buffalo.*

GENTLEMEN:—I shall occupy the present hour with some remarks on the Semeiology of the Eye, to which I invite your usual attention.

The disturbances which take place in the action of the external senses may depend upon an altered state of the organs themselves, or they may be symptomatic of an affection of the brain, or of some distant organ. For the integrity of their function, they depend on the healthy state of the brain; that is to say, a diseased condition of this organ, is incompatible with their perfect soundness; they may, it is true, be perverted, or destroyed, by disease set up in the organs themselves. This is probably more often the case with the organ of hearing than with that of sight. Their function also may be interrupted by a disease of the nerves, which convey the impressions to the brain. Now, which of these four causes, are concerned in individual cases of deranged function, must be judged of from the accompanying signs, and from the absence of those usually connected with the other causes. As means of prognosis, those signs indicate,

---

*It is possible that the phraseology of the following lecture, prepared some time since, may, in a few instances, have been borrowed from other sources, and not marked as quotations at the time; and the authorities have now escaped from recollection. This statement is made to obviate any charge of plagiarism.

of course, most danger, which proceed from the brain itself; and those are least dangerous which proceed from the organs of the senses, or from other remote organs, as dilated pupil, squinting, &c., from the irritation of worms in the intestinal canal.

The functions of the senses may be altered in *four* different ways. They may be *exalted, diminished, suspended,* or *depraved,* and all these changes may occur from either of the above causes.

You will often find an exaltation of the functions of all the senses, in hysterical and hypochondriacal states, and you will find hearing and sight exalted in the commencement of cerebral diseases, and as generally diminished towards their termination; in all acute diseases, it is a good sign if the senses continue in their natural state; or if their functions have been disturbed, that they have again become normal at the time of the crisis, and after it.

There are various changes which take place in the eye, and the faculty of vision, which are important in a semeiological point of view. The eye may undergo a change with regard to its volume, its color, its position, its form, its brightness, its motions, its power of seeing, its internal structure, and its expression. *Morbid enlargement of the eyes*, may arise from *congestion* in the organ, from *hydropthalmus*, from *medullary sarcoma, fungous excrescence, scirrhus* of the eye-ball, *melanosis* of the eye-ball, &c. In *staphyloma pellucidum*, or *conical cornea*, it is only the cornea which is altered in shape, is projecting in the form of a cone more or less acute, producing at first short-sightedness, and afterwards total loss of distinct vision. *Hydropthalmia* or dropsy of the eye, assumes three forms: 1st. Dropsy of the Aqueous Humour, which usually follows blows, or other injuries of the eye, though it sometimes follows suppression of cutaneous eruptions; this is limited in extent, and usually combined with a paralytic and tremulous state of the iris, and partial amaurosis. 2d. *Sub-Sclerotic Dropsy*, which is an accumulation of fluid between the sclerotica and the external surface of the choroid coat, which is relieved by puncturing the eye and allowing the fluid to escape. 3d. *Sub-Choroid Dropsy*, which is an effusion of water between the choroid and the retina, from inflammation of the choroid coat. This disease has been known in several cases to proceed so far as to cause the absorption of the Vitreous humour, and the compression of the retina into a cord, extending from the optic nerve to the back of the lens. The accumulation of water is usually slow, as is also the loss of vision; but in some cases the effusion is rapid, causing great pain in the eye and head; the choroid pressing against the sclerotica produces the extenuation of the latter, while the eye-ball undergoes either a general or partial enlargement; and the pupil becomes dilated and sometimes displaced. The treatment here, is to puncture the eye at the usual place of introducing the cataract-needle. 4th. *Dropsy of the Vitreous Humour.* Here the increase in the size of the eye may be chiefly in the posterior part of the eye-ball; the eye assuming the form of a cone, the cornea being pushed forward without undergoing any other change, or the aqueous humor may be diminished in quantity, and the iris pushed forward into contact with the cornea,—the iris not being changed in color, nor the pupil extremely dilated. In this affection the sclerotica generally assumes a deep blue color, short sightedness first appears, then complete amaurosis; the eye becoming extremely hard, and its movements sooner impeded than in aqueous dropsy. The pain in the eye and head is intolerably severe from the commencement. The pressure of the enlarged eye-ball, on the walls of the orbit, sometimes induces caries. 5th. *General Dropsy of the Eye.* Sometimes we observe both the aqueous and vitreous humor increased in quantity at the same time, so that the whole eye is greatly enlarged; attended with excessive pain, loss of motion of the eye, loss of sleep, appetite, and at last, of reason. The patient generally dies, worn out with fever, before the eye gives way. Evacuation of the contents, and extirpation are the remedies. The eye is sometimes enlarged from sanguineous effusion into the aqueous chambers, from a blow on the eye, there is also an internal hemorrhage, arising from causes, not well understood, called *apoplexy of the eye.* Enlargement of the eye sometimes proceeds from *fungous excrescence of the iris;* and also from *scirrhus* of *the eye-ball*, *medullary fungus*, *and melanosis.* These are all highly malignant affections and usually prove fatal. I lately witnessed a case of the medullary fungus, where the tumor burst through the fore-part of the eye, advanced with great rapidity, and reached the size of a hen's egg; presenting a spongoid or fungous texture, and attended finally by copious hemorrhage. The patient was a fine boy of eight years old. The disease proved fatal in about six months; and on examining the tumor after death, I found it destitute of fibres, of a brownish, white substance, and having very much the appearance of brain This disease was called *spongoid inflammation*, by Prof. Burns, *fungus hæmatodes*, by Mr. Hey, *medullary sarcoma*, by most writers, and *soft cancer* by Mr. Abernethy. The disease is invariably fatal, I believe, whether extirpation is performed or not, and Mackenzie states that it has resisted both external and internal medicines. *Melanosis of the eye-ball*, is a rare affection, but the disease is readily recognized from the black color of the tumor.

Sometimes the eye appears preternaturally large, in consequence of an effusion of blood, or serum, in the adipose substance of the socket. I lately attended such a case, in a boy who run his head against a post, striking just above the orbit. In six hours the eye was protruded to the extent of nearly half an inch, no other unnatural appearance was visible, and the boy recovered entirely in a few days. You see the eye also protrude very often after the operation for *strabismus*. This is owing not only to a division of the tendon of the internal oblique muscle, but especially to cutting the conjunctival coat over too large a surface, which per mits the eye to protrude, it being this muscle which confines the eye chiefly to the orbital cavity. You may easily be deceived in these cases without careful examination; for a protrusion of the eye is often mistaken for an increase in its size. Where the protrusion is gradual, and permanent, it may arise either from fatty and other tumors developed behind the ball of the eye, aneurism, exostosis, osteo-sarcoma, or inflammation of the adipose tissue in the socket, or general inflammation of the ophthalmic cavity. When the protrusion happens from these causes, the eye is generally pushed to one side. In cases of great determination of blood to the head, the eyes are considerably more prominent than at other times, and this is strikingly the case in apoplexy, paralysis, and even in hysterical paroxysms.

The eyes are sometimes morbidly diminished in size. This may happen from atrophy, suppuration, or loss of separate parts of the eye, as the aqueous, or vitreous humour. A sinking of the eye, (commonly called *hollow eye*) occuring gradually, is a sign of *atrophy* of the parts behind the globe, or absorption of the adipose bed, on which it rolls,—you see this in consumption and most chronic diseases, attended with great emaciation; it it usually, a very unfavorable sign. In cases of long fasting, or starvation, it is also a striking symptom. Where it occurs rapidly, as in cholera asphyxia, in which it is strongly marked, after excessive evacuations, either by blood-letting or from the operation of hydragogue catharties, it denotes a rapid diminution in the serous portion of the blood, and is a symptom of great value in cholera, as well as cholera infantum. In these diseases the eye actually seems to have retreated into the socket, a space, equal, at least, to that of its own diameter. In fevers, according to some of the German pathologists, this sinking is a fatal sign; but I am satisfied this is erroneous. Any cause which produces general emaciation, will produce a sinking of the eye in the same proportion; and the value or significance of the sign, is to be estimated entirely from other circumstances.

The *color* of the eye affords a valuable diagnostic symptom. A red color of the conjunctiva, is an ordinary symptom of inflammation of this coat; and the local character of the complaint is readily determined by the other symptoms, or circumstances attending the case. It is also a common symptom in hyperæmia, and congestion of the brain, and here its diagnostic import is to be determined by the intensity of the cerebral symptoms. In apoplexy, epilepsy, and other affections, where there is a preternatural flow of blood to the head, you will also find the conjunctiva injected, and a blood-shot eye is the common result of a blow upon this organ. A yellowness of the eye is usually associated with that of the skin, and may be occasioned by an absorption of bile into the blood, or the non-separation (owing to a check to the biliary secretion) of those elements which go to the formation of bile. The latter is probably a cause of jaundice in infants.

A *dirty brown color* of the conjunctiva, is apt to occur in malignant fevers of a typhoid type. In bad cases of small pox and measles, and in gangrene, it yields, almost invariably, an unfavorable prognosis. You sometimes observe a fine rose-red vascular net-work under the conjunctiva —this is a sign of *sclerotitis*, and is often of rheumatic origin. An injected state of the vessels of the cornea, are signs of *ceratitis*. White opaque spots on the *cornea*, are the result of inflammation; this may terminate in small *ulcers*, by the healing of which, *cicatrices* are formed. In *iritis*, you observe through the pupil, yellow specs, or exudations of lymph on the iris; and if they be of a dirty grey color, and covered with small red, or yellowish grey elevations, you may be pretty sure that it is of a syphilitic character.

Where there is suppuration of the internal eye, you will see whitish purulent matter, through the pupil, floating in the aqueous humour. A red or dirty grey turbidness in the bottom of the eye, is one of the early symptoms of *medullary sarcoma*; while in *incipient cataract* you observe the crystalline lens of a yellowish white, or ash grey color; whilst greenish turbidness indicates *glaucoma*.

The form and size of the *pupil*, are of considerable importance in the diagnosis of cerebral diseases. A *contracted pupil* generally indicates hyperæmia, or inflammation of the brain, or its membranes, or both; and it may arise from idiopathic or symptomatic affections of this organ. A dilated pupil, although often the result of sympathetic cerebral irritation, as from worms, gastric derangement, hysterical and epileptic fits, is generally produced by pressure upon the brain, either by the effusion of blood or serum, as in hydrocephalus, &c. Such a dilated condition of the pupil is not, however, always the result of pressure, for we find it often in apoplexy. It is a common symptom, also, of narcotic poisoning. The influence of Belladonna in dilating the pupil is well known, and when we wish to produce the full effect of this remedy, we direct it to be given or rubbed around the eye, until there is considerable dilitation of the pupil. You will distinguish contraction of the pupil proceeding from cerebral irritation, from that caused by *iritis* by the following circumstances:—1st. In *iritis*, the pupil is not only contracted, but it is irregular in shape, and immovable. 2nd. There is a beautiful radiating zone of vascular redness, situated in the sclerotica, and immediately surrounding the cornea. 3d, There is a decided change in the color of the iris, from grey or blue to a yellow or greenish tint; and from brown or hazel to a dusky reddish hue. 4th, Closure of the pupil by lymph, unless prevented by active antiphlogistic measures: with the liberal use of calomel. 5th, Dimness of sight, and pain in the eye.

The *lustre of the eye* is closely connected with the state of the brain. You will observe in children, just previous to their being attacked with acute disease, particularly of the brain, (and I have frequently observed the same in croup,) the eye assumes an unnatural brilliance, it fairly sparkles with light, and at the same time, there is great exhiliration of spirits, an exuberance of animal life, which tends to overleap its customary bounds. Parents have often remarked this in their children, though they are, for the most part, ignorant of its true indication. I know many families, where, after it has been pointed out, I have no doubt an acquaintance with the fact has often prevented serious disease, by leading to the early employment of preventive medication. You find the same unnatural lustre of the eye in most cases of hyperæmia and inflammation of the brain, in apoplexy, and in delirium and insanity. Indeed, it is one of those diagnostic marks, by which the experienced eye detects mental disease, where the unpractised observer would discover nothing unnatural. The lustre of the eye fails in states of great prostration of the nervous system, as well as failure of the general strength; hence it is observed in cases of cholera, and cholera infantum, of consumption, low fevers, and poisoning, especially by the acrid mineral poisons. In these low states, you often see the eye covered with a dusky film, which the common people suppose is the infallible precursor of speedy death; but though it is a very unfavorable sign, it is by no means a fatal one, as I have repeatedly observed.

The *motions of the eyes* are regarded by the pathologist, as affording valuable diagnostic and prognostic aid. The eye is moved, as you know, by six voluntary muscles; four straight, a superior, inferior, external and internal; and two oblique, a superior, with its tendon running round a pully, and an inferior. The straight muscles are for the direction of the eyes, and we become acquainted with the distances, magnitudes and positions of objects, by the sensations which accompany the motions of the muscles of the eye, or in other words, from the consciousness of muscular effort. (*Elliotson.*) Immobility of the eyes is a sign of tonic spasm, or paralysis of the muscles of the eyes, and often, of course, indicates cerebral disease, as effusion of serum or blood, or violent congestion; it is also observed in epilepsy and catalepsy. Too violent movement of the eyes is a sign of clonic spasm, and is a common symptom in acute affections of the brain, as apoplexy, in cases of delirium, and general spasm. Diminished mobility is observed in diseases of great prostration, as typhus fever, cholera, poisoning by arsenic, &c.

Squinting, *strabismus*, may be permanent, when it is owing to a contraction of one of the muscles of the ball, which is now so easily remedied by the operation of myotomy; or temporary, when it is involuntary, and caused by cerebral disease, as hydrocephalus, apoplexy, encephalitis, &c. In such cases, of course, it affords an unfavorable prognosis, but it is also a symptom of worms, and is not unfrequent in hysterical and epileptic paroxysms.

The motions of the iris have the same importance as those of the eyeball, and may result from different causes, both local and general. Its motions are dependent on the third pair of nerves, and when these are divided, the pupil dilates and cannot be made to contract by the most intense light. A fixed iris is the result of tonic spasm, or of paralysis; these may be local, or owing to congestion, or inflammation of the brain. Its contractibility is lessened in all low typhoid states of the system, while it is increased by the opposite condition. The *look* or *expression* generally harmonizes with the features; thus there is a wild look, an anxious look, and a despairing look. A wild expression indicates irritation, hyperæmia or inflammation of the brain; it is generally associated with delirium, and precedes apoplexy. You see an anxious look in all diseases of the heart, and generally wherever there is much obstruction to the respiration or circulation. The despairing look belongs to violent diseases of the digestive organs, as gastritis, enteritis, acute hepatitis, cholera and dysentery.

*Defective Vision.* There are various states of defective vision, such as 1, *myopia*, or *near-sightedness.* This may be caused by, 1, too great convexity of the cornea; or, 2, too great thickness of the cornea; or 3d, too great convexity of the crystaline lens; or 4, preternatural density of any, or all of the transparent media of the eye; or 5, preternatural elongation of the eye-ball.

2. *Presbyopia* or far-sightedness. The cause of this is deficient refraction, owing to too great flatness of the cornea, from diminution in the quantity of the aqueous and vitreous humours; or diminished density in any of the refractive media of the eye. The density of the crystalline lens increases with age, but it probably shrinks so as to become less convex. When presbyopia occurs suddenly in subjects much under the age of 40 years, we should be led to suspect, either some derangement of the internal parts of the eye, some pressure behind the eye-ball, or some disease of that portion of the optic apparatus which is contained within the cranium. In some individuals, there is an insensibility to certain colors; they cannot, for example, distinguish *red* from *green*, or *pink* from *blue; red* appearing to them merely a *dark* color, while *green* is a shade of *drab.* They can easily distinguish *yellow* and *blue*, but they judge of *orange, purple* and *brown* with great difficulty; and even the shades of *black, gray* and *white*, they are often unable to decide upon without hesitation. At least five different explanations have been given of this phenomenon; but it seems to me that that furnished by phrenology is the most satisfactory, and this is that the faculty of distinguishing colors depends on a definite portion of brain, called the *organ of color;* and that defects in this faculty must be attributed to this organ, and not to the eye, which is apparently perfect.

*Colored vision* is not an unfrequent symptom of cerebral derangement. In perfect health, we, of course suffer no imitations of visual sensations, no flashes of light from internal changes in the eye, no false perceptions of *muscæ volitantes*; objects are seen clothed in their natural colors, and no objects are supposed to be seen, except when their image is actually impressed upon the retina. And, yet, we are subject to false visual sensations of various kinds and degrees. When the circulation of the blood is accelerated through the retina and neighboring parts, we can actually see the circulation of the blood in the eye; thus if we look on a white wall well illuminated, we see a kind of net-work, darker than the other parts of the wall, which appears and vanishes alternately, with every pulsation. This change of color is owing to the compression of the retina by the diastole of the artery. Dr. R. Darwin states that we can see the circulation in the eye at any time, by rubbing the eyes well, and holding the breath so as to accumulate more blood in the head, then turning to the light, the small veins and arteries are distinctly seen. In general, however, the circulation in the eye may easily be seen by examining other spectra with your back to the light till the eye becomes weary, then having covered your closed eye-lids for half a minute, till the spectrum is faded away, which you were examining, turn your face to the light, and removing your hands from the eye-lids, by and by shade them again a little, and the circulation becomes perfectly distinct. It is probably the veins which we see, as the blood in them is darker, and they seem to unite after the usual manner of veins. You will sometimes hear patients complain of the sensations of seeing luminous objects, like a lighted candle, surrounded by the colors of the rainbow, or luminous flashes or sparks. This is a very frequent symptom in amaurosis; it is also present in inflammation of the eye, causing a derangement of the retina, but it may be owing to some derangement of the lenses of the eye, by which the achromatic power of this organ becomes impaired. The same effect may be produced in a degree, by contracting the eye-lids, by which the rays of light are decomposed by inflection merely.

Sometimes persons complain of seeing objects tinged of a yellow, green or bluish color. Thus in jaundice, patients often see objects colored yellow; if blood is effused into the anterior chamber, every thing appears red. This phenomenon has been called *Chrupsia*, from *Kroma*, color, and *Opsis*, vision.

There is a frequent morbid phenomenon, connected with the eye, which has been called *Photopsia*, from *Phos*, light, and *Opsis*, vision. It is sensations of light, independent of vision, as that produced by sneezing, or a sudden blow on the eye, or by galvanism or electricity, as in the well known experiment of applying a piece of zinc and a piece of silver to the tongue, and then bringing them into contact. If any experiment were needed to show that the retina may be so impressed as to produce the sensation of light altogether independently of the actual presence of light, we have only to rub our eyes with the finger, or produce slight pressure on the globe, when flashes of light will instantly appear. In *retinitis*, and also some kinds of *amaurosis*, at their commencement, there will be flashes of light, the appearance of shining stars, a glittering as if from the points of innumerable needles, and a variety of other lucid spectra; and in some peculiar cases, the patient complains of seeing globes of light, swimming before him, or as if he were looking at a sea of melted gold. These lucid spectra should never be disregarded, or thought lightly of, for they are very often the precursors of convulsive attacks, such as epilepsy; persons inclined to apoplexy, often see showers of shining spectra on raising their heads after stooping; the same appearances attend phrenitis, and cephalitis, and they are particularly troublesome to those who have suffered from internal ophthalmia. The treatment, of course, is to be regulated entirely by the cause.

*Ocular Spectra*, or *accidental colors* are not unimportant to the pathologist. If you look at a bright object for a short time, and then close your eyes, you see the form of the object, but generally of a different color; this is called the *ocular spectrum* of that object. Buffon gave to colors arising in this way, the name of *accidental* colors, to distinguish them from those which are produced by the decomposition of white light. The causes of this phenomenon are not well understood, but when you find persons complaining of ocular spectra for days, and weeks together, you may expect that it will be followed by some serious affection of the retina. Dr. Brewster, of Edinburgh, nearly lost his sight by the following experiment: On a bright, clear day, when the sun was near the meridian, he formed a very brilliant and distinct image of its disc, by means of the concave mirror of a reflecting telescope. His right eye being tied up, he viewed this luminous disc with the left, through a tube, which prevented any extraneous light from falling upon the retina. When the retina was highly excited by the solar image, he turned his left eye to a white ground, and examined the series of ocular spectra which followed. After uncovering the right eye, a remarkable phenomenon appeared; for on turning it on a white ground, he found that it gave also a colored spectrum. He repeated the experiment twice, in order to be secure against deception, and always with the same result. The spectrum in the left eye was uniformly invigorated by closing the eye-lids, because the images of external objects efface the impression upon the retina, and when he refreshed the spectrum in the left eye, that in the right was also strengthened. On repeating this experiment a third time, the spectrum appeared in both eyes, which seems to prove that the impression of the solar image was conveyed by the optic nerve from the left to the right eye; for the right eye being shut, could not be affected by the luminous change. The effect of this experiment was to reduce the eyes to such a state of extreme debility that they were unfit for any further trials. A spectrum of a darkish hue floated before his left eye for many hours, succeeded by the most excruciating pains, shooting through every part of the head. These pains, accompanied with a slight inflammation in both eyes, lasted for several days. Two years after, the debility of the eyes still continued, and several parts of the retina in both eyes had completely lost their sensibility. (*Ed. Cyclop.*)

You may perform the experiment with perfect safety, by placing a *red wafer* on a sheet of white paper, and fixing the eye for some time steadily to a dot at its centre, then turn the eye to another portion of the paper, and you see a circular spot of the same size as the wafer, but its color will be *green.* You will bear in mind that there are but three primary and distinct colors, *red, yellow* and *blue,* all the other colors being compound or composed of a combination of these. Thus, *orange* is a compound of *red* and *yellow; green* of *yellow* and *blue; purple* of *blue* and *red;* and *white* is a neutral combination of the three primary colors. An *accidental* color is always that, which added to the original color, produces *white.* Thus if you look at the *red* wafer, the spectrum is *green;* this is the result of the mixture of *yellow* and *blue,* but *yellow* and *blue,* with *red,* go to form white; these are therefore called *complimentary* colors, and so of the others.

*Muscæ Volitantes.* These are false visual sensations, which have derived their name from their resemblance which they bear to *flies* moving through the air. These differ much, however, in appearance, some being like twisted tubes, partially filled with globules, which sometimes appear in motion, while others look like black spots which follow the motions of the eye.

Mackenzie thinks that the *black spots,* which he compares to the body of a spider, with three or four diverging legs, are often the precursor of *amaurosis,* while the semi-transparent spectra portend nothing serious. I have, however, known the black spectra continue for years without being attended by any serious inconvenience, and finally disappear altogether. Dr. Mackenzie thinks that *muscæ* volitantes are never seen in the sense in which objects out of the eye are seen, "as," he remarks, "opaque spots in any part of the eye, anterior to the retina, could never produce an image on that membrane, sufficiently defined to give rise to such impressions as the generality of muscæ volitantes; he, therefore, is inclined to refer them to the retina itself, the semi-transparent being caused by a dilation of the branches of the *arteriæ centralis retina,* while the dark spots are the effects of certain portions of the retina having become insensible to light." Mrs. Griffith, of New York, who has writen an excellent work on the eye, believes them to be *air bubbles,* floating in the liquor which surrounds the crystaline lens, and is collected within its capsule. She maintains that this phenomenon is neither the cause, nor the consequence of *amaurosis,* but is occasioned solely by a debility of the absorbent vessels of the part. "These bubbles," she remarks, "when single, or even when united like strings of beads, do not obstruct the rays of light, because their axes are independent of each other. But when they cling together in bunches, their axes interfere, and do not allow the rays of light to pass through them, consequently they present as great an obstruction as any other opaque mass. It is simply, therefore," she continues, "through the want of parallelism in the axis of these air-bubbles, that the spectra called *muscæ volitantes,* disturb our vision." (*N. York Lancet, vol.* 2, *p.* 212.)

There are few symptoms which prove so alarming to nervous people as these appearances in the eye. They often suppose they are about to lose their sight by cataract, or amaurosis. But you may very safely assure them that there is no danger of either of these terminations, unless other symptoms be present. These false perceptions, for so I regard them, do not render objects obscure, as incipient cataract does; nor is there any fixedness or even unnatural slowness of the iris, in simple cases of *muscæ volitantes.*

*Spectral Illusions.* Hippocrates has noticed the fact in his work, "*De Natura Muliebri,*" that spectral illusions are often attended by fatal effects. Dr. Hibbut, in his excellent work entitled "*Sketches of the Philosophy of Apparitions,*" has traced them to a great variety of causes, such as *highly excited states of particular temperaments, hysteria, hypochondriasis, the neglect of accustomed periodical blood-letting, febrile and inflammatory affections, inflammation of the brain, delirium tremens, &c.*

These spectral illusions are almost infinite in variety and form, and we may assure those who are troubled with them, that they are merely disordered sensations, symptoms of a peculiar affection of the internal optic apparatus, the effect of which is, to produce a repetition or imitation of former impressions.

Mr. G. Combe thus explains the existence of spectral illusions: "When any of the knowing or reflecting organs is internally active, the mind conceives, or is presented with ideas of the objects which it is fitted to perceive. Thus, locality, coloring, and size being active, we are able, with our eyes closed, to conceive a landscape, in all its details of hill and dale, sunshine and shade. If this internal activity become morbid, through disease of the organs, their ideas become fixed, and remain involuntarily in the mind: and if this is long continued, it constitutes insanity. Many persons have experienced, when in the dark, vivid impressions of figures of every variety of color and form passing before the mind, sometimes invested in alarming brilliancy and vivacity. I conclude that this arises from an internal excitement of the organs situated at the superciliary ridge, viz: form, locality, coloring, &c., occasioned generally by an unusual accumulation of blood. This affection is, in most cases, only momentary; but suppose that it were to become fixed and continuous, then the mind would be haunted with permanent and vivid conceptions of innumerable and fantastic beings, invested with more than the forms and hues of realities. This would be insanity; not a diseased feeling, such as melancholy, a fury, or religious joy, but an intellectual delusion, so that every sentiment might be sound, and yet this aberration of intellect remain fixed and immovable by the will." This, gentlemen, appears to me a much more satisfactory and philosophical explanation, than that usually given.

By this means, we shall be able to calm the minds of some who otherwise might be led to ascribe their visions to supernatural powers, or who through fear or terror might possibly be driven to insanity.

*Night Blindness* is a rare affection, but one that we sometimes meet with, and it is one which always gives great alarm. Without, perhaps, any premonitory symptoms, a person finds himself all at once, as night comes on, nearly blind. The physician is immediately sent for, but he finds nothing to be seen, except a dilatation of the pupils. He bleeds the patient, probably, and gives him a cathartic, and in the morning, to his surprise, he finds himself perfectly well. At evening, however, his blindness again returns; if candles are present, they look as if glimmering through a thick mist or fog. Thus the complaint goes on, returning at night, disappearing by day, till after a while, the vision becomes indistinct by day, as well as by night, though not in so great a degree. The patient grows near sighted, and finally, if neglected or mistreated, the complaint ends in incurable amaurosis. Dr. Mackenzie thinks the cause of this disease is in some peculiar state of the choroid coat, rendering the eye insensible to light of a certain intensity. But I am inclined to locate the disease in some peculiar modification of that portion of cerebral substance, in which the optic nerve originates, for it is a well known fact that the disease is frequently sympathetic of derangements of the digestive organs, as shown by the foul tongue, fetid breath, and deficient appetite. It is also a precursor of scurvy, and in the East Indies, is supposed to be caused by eating hot rice. It is, however, owing in some instances to exposure to an unusual glare of light, especially in warm climates, or sleeping with the eyes exposed to the sun's rays. The treatment is to be regulated by circumstances, but as a general rule, it must be constitutional.

In some instances, as after being long shut up in a dark room or prison, the pupil becomes so much dilated by exposure to the light of day, that the patient is unable to see—this is called *mydriasis*; at night, as the pupil contracts by the withdrawal of the light, vision becomes restored. Again, the pupil may contract so much by day, as to obstruct the sight; but as it relaxes in the dark, the person is again enabled to see. For the same reason, a person laboring under incipient cataract can see better during the evening, than during the brightness of the day, for then the pupil dilates so as to permit more light to reach the retina. This is called *Day-Blindness*. Baron Larrey relates the case of an old man, one of the galley slaves at Brest, who had been shut up in a subterranean dungeon for the space of 33 years; in consequence of which, he was perfectly blind during the day, and could see only at night. Romazzini records the prevalence of this disease, as an epidemic, among boys about ten years of age, in the month of March, which had disappeared by the middle of April. There is another defect of vision, which goes under the name of *amaurosis*. If the retina be incapable of receiving with correctness, impressions of external objects through the medium of light, or, if the optic nerve be unable to convey to the sensorium the impressions made upon the retina; or, if the sensorium be incapable of receiving the impressions conveyed by the optic nerve, the individual must necessarily be affected with a greater or less obscurity in vision, or suffer a total deprivation of vision, according to the degree of inability in these several parts to execute their functions. Now it is a very important, and sometimes a difficult matter, to distinguish these cases, and pronounce with certainty, whether the retina, the optic nerve, or the brain, be the part first and principally affected. There are numerous anatomical and physiological facts which necessarily enter into a consideration of the seat of the different varieties of this disease; but it is absolutely essential that the physician should be most accurately acquainted with the structure and functions of the nervous system, in order to arrive at any thing like certainty in his diagnosis. It would require two or three lectures to do any thing like justice to this affection; I shall, therefore, merely remark as to its *efficient causes*, that they may be 1st, of a *local*,

*direct*, and *mechanical* nature, such as the pressure of a tumour on the optic nerve; or 2d, of a *local* and *vital* kind, such as a plethoric or congested state of the blood vessels of the brain, or of the eye; and 3d, the cause has been *general* or *constitutional*, such as exhaustion, from blood-letting, or excessive loss of any of the fluids of the body. Its *proximate* cause in a large number of cases, at least, is *pressure*. The *remote* causes are numerous; as hereditary predisposition; over-exertion of the sight, and exposure to too great light; all causes which tend to produce cerebral congestion—poisons, especially those of narcotic vegetables, and especially tobacco; (according to Mackenzie,) gastric and intestinal irritation; exhaustion of the body from any cause whatever, particularly grief, excessive venery, and protracted suckling; blows on the head, &c. These hints, brief as they are, will serve to show that the subject is surrounded with difficulties, and that there is the greatest necessity for exercising the most minute and careful observation, if we hope to make any advancement in the knowledge of this class of diseases. Our prognosis in this affection will depend entirely on its cause and seat, and these must be accurately determined before we can determine any thing, or form any opinion as to its progress and termination. From what I have observed, I am inclined to believe that the cause of amaurosis is oftener seated in the brain than in the eye, hence its obstinacy and general incurability.

*Acute Vision.* The sight often becomes morbidly acute, 1st, in some cases of inflammation of the brain or its membranes; in nervous fevers; in mania, and in hyperæmia of the brain from acute inflammatory affections of other organs. 2d. In diseases where the sensibility in general is exalted, as hysteria, and hypochondriasis, and in recovery from acute diseases. 3d. In inflammation of different parts of the eye, more especially in catarrhal inflammations, in variola, rubeola, scarlatina, scrofula, and arthritis, and in inflammation of the iris, of the capsule of the lens, and of the retina. Preternatural acuteness of vision often precedes attacks of acute diseases of the brain, as in the case related by Dr. Brachet, of a man in one of the surgical wards of the Bicetre Hospital, at Paris, who suddenly acquired such an astonishing acuteness of sight, that he could see the most minute objects at an enormous distance. Five hours afterwards he felt a slight headache, and in a few hours more was seized with a fatal apoplexy. On dissection, a fresh coagulum of blood was found in the right optic thalamus. The inflammation which had preceded the effusion, had irritated by its proximity that portion of the brain from whence the optic nerve takes its origin. I have known the same happen both to the organ of sight and hearing, from an attack of hemiplegia; the increased acuteness of both these senses was in the highest degree astonishing.

*Double Vision* is not unfrequently met with, and it usually proceeds from irritation, inflammation, or effusion upon the brain; it is a common symptom in hysteria, hypochondriasis, worms, intoxication, delirium tremens, &c. Where it proceeds from an affection of the retina, it is a sign of impending amaurosis. In acute diseases, and in gout, it is a sign of metastasis to the brain; if it occurs in affections of the spinal cord, it renders our prognosis more unfavorable by showing a complication with cerebral disease. In *deformed vision*, that is, where one sees objects disfigured, inverted, or crooked, there is much reason to suspect organic disease of the brain.

These are all the remarks which my time will allow me to make on the interesting subject of the eye, especially with reference to its *Semeiology*. I have dwelt the longer upon it because, though one of the smallest organs, it is nevertheless one of the most important in the body; and especially because its diseases furnish more satisfactory and plainer illustrations of the general doctrines and facts of pathology, than any other single organ of the body. Here you see, in fact, almost all diseases in miniature; and from the peculiar structure of the eye, you see them as through a glass, and you learn many of the little wonderful details, in the nature of morbid processes, which but for the observation of them in the eye, would not have been known at all.—(Latham.)

